# Development and Holocrine Secretion of Resin Ducts in *Kielmeyera appariciana* (Calophyllaceae)

**DOI:** 10.3390/plants13131757

**Published:** 2024-06-25

**Authors:** Ellenhise Ribeiro Costa, Diego Demarco

**Affiliations:** Departamento de Botânica, Instituto de Biociências, Universidade de São Paulo, São Paulo 05508-090, SP, Brazil; elllenzinha27@gmail.com

**Keywords:** secretory ducts, lumen formation, programmed cell death, secretion release, *Kielmeyera*

## Abstract

The modes of formation and release of secretion are complex processes that occur in secretory ducts and their description has great divergence in some species. The use of modern techniques to detect hydrolytic enzymes, cytoskeleton arrangement and indicators of programmed cell death may help clarify the processes involved during the ontogeny of that gland. The goal of our study was to analyze subcellular changes during schizogenous formation and secretion production and release into the lumen in resin ducts of *Kielmeyera appariciana*. Our results demonstrate the participation of pectinase through the loosening of the central cells of the rosette, which subsequently split from each other through polarized growth mediated by a rearrangement of the microtubules. The resin is mainly synthesized in plastids and endoplasmic reticulum and is observed inside vesicles and small vacuoles. The secretion release is holocrine and occurs through programmed cell death related to the release of reactive oxygen species, causing cytoplasm darkening, chromatin condensation, vacuole rupture and plastid and mitochondria degeneration. Cellulase activity was identified prior to the rupture of the cell wall, causing the release of secretion into the lumen of the duct. The participation of the cytoskeleton was observed for the first time during schizogeny of ducts as well as programmed cell death as part of the process of the release of holocrine secretion. This type of secretion release may be a key innovation in *Kielmeyera* since it has not been observed in ducts of any other plant thus far.

## 1. Introduction

Secretory ducts are glands constituted of an epithelium of secretory cells that delimit an elongated lumen in which the exudate is stored. This exudate has a variable chemical nature and is produced and released into the lumen through various processes [1]. During the development of ducts in plants, two distinct processes are involved in their formation and secretion, although these processes are often confused since the secretory phase usually begins before the duct reaches its final dimensions [2].

The formation mode refers to the structural development of the duct, which may involve the separation of cells (schizogeny), programmed cell death (lysigeny) or both processes (schizolisygeny) [1,2]. On the other hand, the secretion mode (or secretion release mode) is the mechanism through which the compounds produced by secretory cells are transferred to the outside of the protoplast [3]. This process usually occurs without cell disruption and is termed merocrine secretion. In this mechanism, the secretion can leave the protoplast, crossing the plasma membrane by diffusion (eccrine secretion) or by secretion packaged in vesicles and/or vacuoles that fuse to the plasma membrane and release the secretion (granulocrine secretion) [1]. Once in the periplasmic space, the secretion can pass through the cell wall by diffusion or be pushed by the active pressure of the protoplast, depending on the composition and viscosity of the compounds [3]. Merocrine secretion occurs in most secretory ducts, but a second type of mechanism has also been reported for many ducts: the holocrine secretion. In this release process, the secreted substance leaves the secretory cell as a result of its disintegration [1]. This process is relatively common in the secretory ducts of some plants and has also been reported in other types of glands, such as nectaries and salt glands [1,4,5,6,7].

The mechanism of cell lysis to secretion release may be related to a process of programmed cell death (PCD) of the secretory cell [8]. This process is common and occurs in several stages of plant development related to various tissue functions during leaf formation, root cap and anther development, ovule fertilization, fruit maturation, seed formation and germination and the well-known tracheary element differentiation [9,10]. PCD is usually identified by cytoplasm darkening and nuclear chromatin condensation, followed by cell wall loosening and tonoplast and plasma membrane rupture, resulting in cell death [11]. It is not yet known what all the signaling mechanisms are that are behind the cell death process, but it is known that the process of producing reactive oxygen species is involved in the activation of this process [9,12,13].

All these events culminate in the lysis of the secretory cell, releasing the secretion. The secretory compounds at times have medicinal properties, and the secretion is used in folk medicine. Several species of Calophyllaceae produce secretions with anti-inflammatory, antioxidant, antibacterial and antifungal properties, and microbiological tests have confirmed the efficacy of some compounds against cancer cell lineages [14,15,16,17,18]. Among these medicinal species is *Kielmeyera appariciana*, which stands out for having resin ducts in the primary shoot and gum ducts in the bark [19].

Resin ducts of *K. appariciana* have been described as schizogenous, but there are no data on cellular changes related to the separation of cells or to the process of secretion release into the lumen [19]. Our knowledge of the action of enzymes and cytoskeleton in gland development and secretion release is still incipient, with studies carried out in ducts, glandular trichomes and laticifers of very few species [20,21,22,23]. In light of the scarcity of data and the many questions yet to be answered, the objective of this work was to analyze the development of the resin ducts of *K. appariciana* and the processes involved in the synthesis and release of the secretion.

## 2. Results

### 2.1. Development and Secretion Mode of Resin Ducts

**Figure 1:**
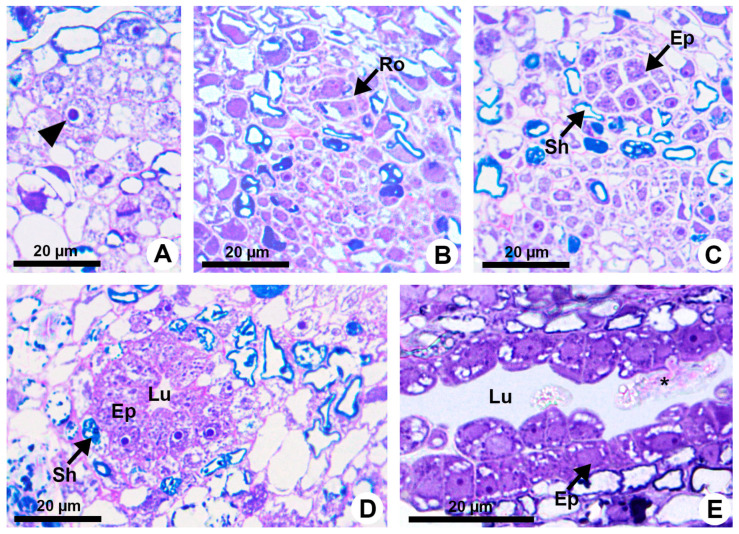
Resin duct development in *Kielmeyera appariciana* Saddi. (**A**–**D**) Transverse sections. (**A**) Initial cell of the ground meristem (arrowhead) from which the duct will originate. (**B**,**C**) Sequence of divisions of the initial cell, giving rise to the rosette. (**D**,**E**) Mature duct in the secretory phase. (**E**) Longitudinal section of the duct. Note the release of the disintegrated protoplast of epithelial cell (asterisk) into the lumen (Ep = epithelium; Lu = lumen; Ro = rosette; Sh = sheath).

**Figure 2:**
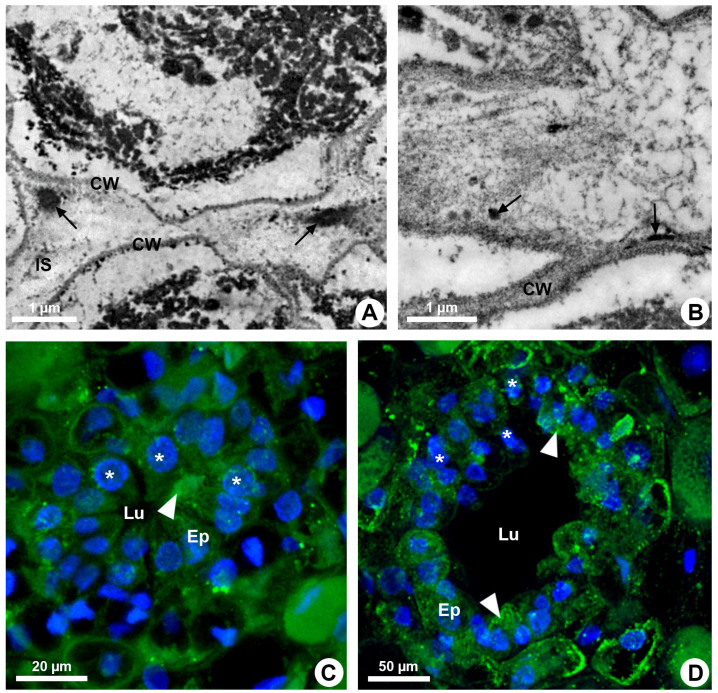
Enzymatic activity and microtubule organization in developing resin ducts of *Kielmeyera appariciana* Saddi. Transverse sections. (**A**,**B**) Transmission electron microscopy. (**A**) Pectinase activity detected in the middle lamella (arrow). Note the emergence of a narrow intercellular space. (**B**) Cellulase activity detected in the cell wall of the epithelial cells (arrow), facing the intercellular space. (**C**,**D**) Immunolocalization of microtubules (green) and DNA labeling (blue) under confocal laser microscopy. Note the polarized organization of microtubules in some epithelial cells (arrowhead) and nuclei with regions of condensed chromatin (asterisk) (CW = cell wall; Ep = epithelium; IS = intercellular space; Lu = lumen).

Resin ducts of *K. appariciana* originate from a single cell of the ground meristem (Figure 1A). This initial cell has thin walls, dense cytoplasm and a prominent nucleus (Figure 1A). Successive divisions of this cell form a rosette of still undifferentiated cells (Figure 1B) that remain in constant division, later differentiating into the epithelium and sheath (Figure 1C). The lumen of the duct is formed by schizogeny (cell separation) without signs of cell lysis in the early stages of duct development (Figure 1D,E). The cytochemical analysis identified pectinase activity in the middle lamella between the central cells of the rosette (Figure 2A), loosening them and forming a narrow intercellular space by dissolution of the middle lamella. At the same time, cellulase activity is observed in the portion of the cell wall of the developing epithelium facing this intercellular space (Figure 2B). Then, some points of greater concentration of microtubules are observed in polarized regions of the epithelial cells (Figure 2C) and they start to split away, expanding the intercellular space and forming the lumen (Figure 2C,D). While the ducts develop and expand their lumen, the epithelial cells are constantly producing secretion (Figure 1D,E), which is temporarily stored within the cell. Mature ducts have a uniseriate secretory epithelium, constituted of thin-walled cells with dense cytoplasm and prominent nuclei (Figure 1D). The ducts are axially elongated (Figure 1E) with a uni- or biseriate sheath formed by cells with phenolic compounds (Figure 1C,D) and can vary in diameter and depth (Figure 1D and Figure 2D).

**Figure 3:**
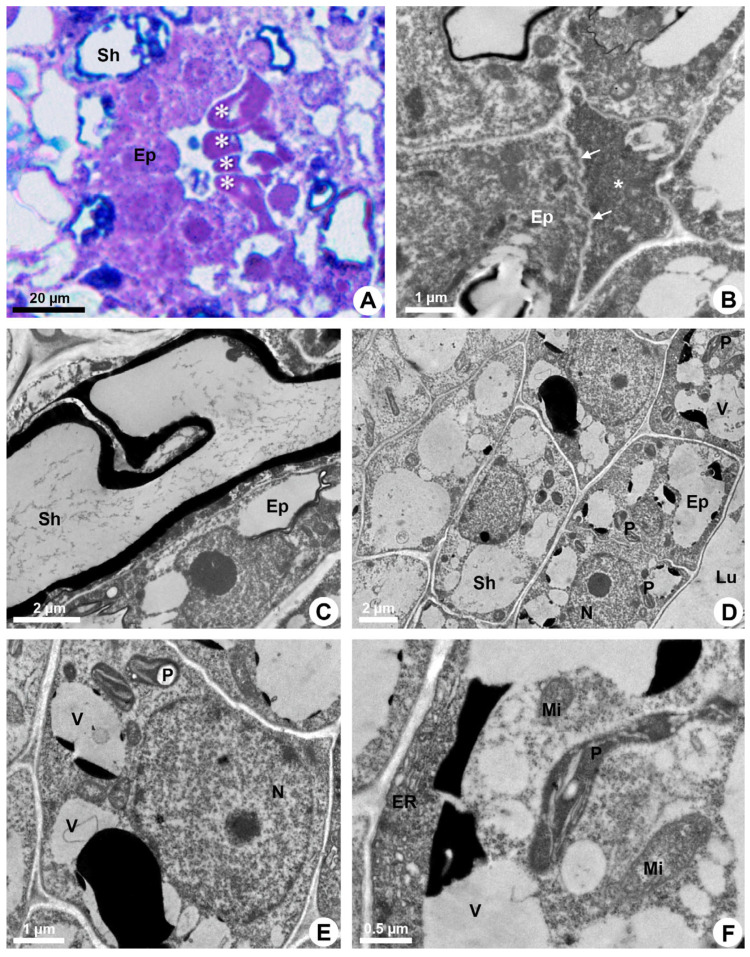
Secretory process in resin ducts of *Kielmeyera appariciana* Saddi. (**A**) Light microscopy. Bright field. (**B**–**F**) Transmission electron microscopy. (**A**,**B**,**F**) Transverse sections. (**C**–**E**) Longitudinal sections. (**A**,**B**) Mature ducts. (**A**) Epithelial cells in programmed cell death (PCD) evidenced by darkened cytoplasm (asterisk). (**B**) Degenerated protoplast of an epithelial cell (asterisk) in PCD. Note a living epithelial cell with a shrunken periclinal cell wall (arrow). (**C**–**F**) Epithelial cells during the secretory phase. Young ducts. (**C**) Epithelial cell with a voluminous nucleus and evident nucleolus. Note the sheath cell with a large vacuole containing phenolic compounds. (**D**,**E**) General view of epithelial cells. Note the presence of numerous elaioplasts and secretory vacuoles. (**F**) Epithelial cell with parietal endoplasmic reticulum, large elaioplast, mitochondria and secretory vacuoles (Ep = epithelium; ER = endoplasmic reticulum; Lu = lumen; Mi = mitochondrion; N = nucleus; P = elaioplast; Sh = sheath; V = vacuole).

During the secretory phase, epithelial cells are elongated (Figure 1D) and signs of cell death are observed in some of them, evidenced by dark-stained cells (Figure 3A). This process is asynchronous and only some cells show cellular disintegration in each region of the epithelium. DAPI assay also demonstrated that the nuclei of some cells showed a loss of shape, becoming more elongated and with many regions of heterochromatin (Figure 2C,D). Finally, these cells rupture and release their contents into the lumen (Figure 1E and Figure 3B).

### 2.2. Subcellular Secretory Machinery and Holocrine Secretion

Mature ducts have secreting cells with lumen filled with a heterogeneous exudate (Figure 2A). Epithelial cells have ribosome-rich cytoplasm, numerous plastids and several vacuoles of different sizes, besides abundant endoplasmic reticulum, some dictyosomes and voluminous nucleus with decondensed chromatin and evident nucleolus (Figure 3C–F).

**Figure 4:**
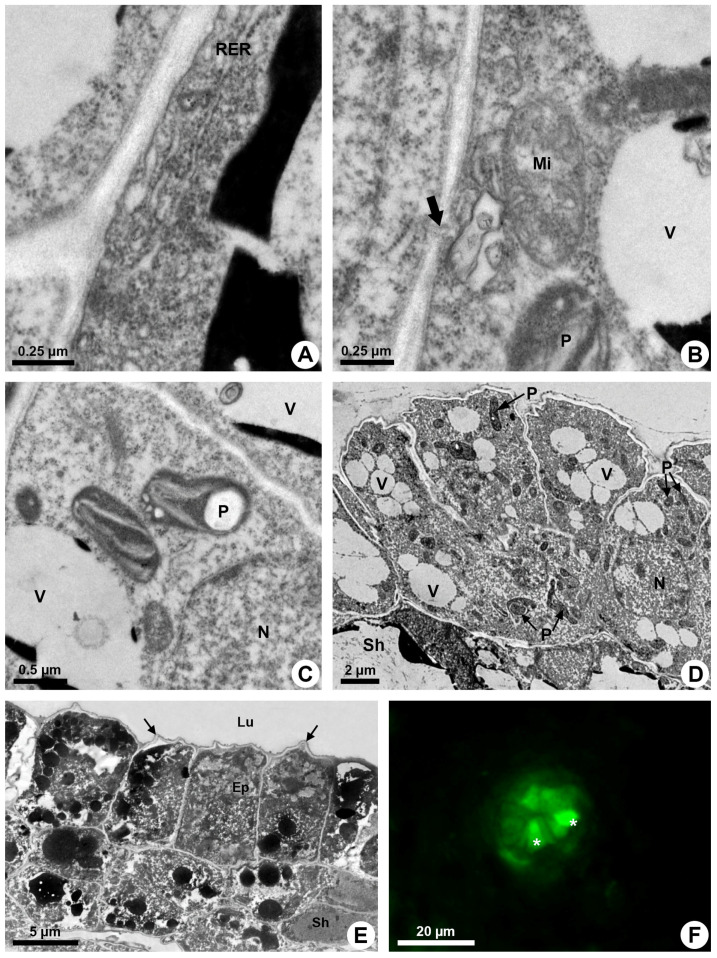
Secretory process in resin ducts of *Kielmeyera appariciana* Saddi. (**A**–**E**) Transmission electron microscopy. (**F**) Fluorescence microscopy. (**A**–**D**) Young ducts. (**E,F**) Mature ducts. (**A**) Epithelial cell with parietal rough endoplasmic reticulum. (**B**) Mitochondrion with distinct cristae near cell wall, where a plasmodesma is observed (large arrow). (**C**) Elaioplasts with few wide thylakoids. Note the presence of a large starch grain. (**D**) General view of the epithelium with cells containing many elaioplasts and dispersed vacuome. (**E**) Epithelial cells containing many vacuoles and vesicles with electron-dense material at the end of the secretory phase. Note the shrunken periclinal cell walls facing the lumen (narrow arrow). (**F**) ROS detected in epithelial cells (asterisk) treated with DCFH-DA (Ep = epithelium; Lu = lumen; Mi = mitochondrion; N = nucleus; P = elaioplast; RER = rough endoplasmic reticulum; Sh = sheath; V = vacuole).

The endoplasmic reticulum is especially extensive close to the plasma membrane and is associated with ribosomes (RER) in some areas (Figure 4A). Elaioplasts with few wide thylakoids containing starch grains (Figure 4B,C), small mitochondria with distinct cristae (Figure 4B) and vacuoles with secretion (Figure 3E,F) are prominent during the secretory phase (Figure 4D).

The secretion is mainly produced by elaioplasts and endoplasmic reticulum that show dilated cisterns in which secretion is accumulated. Many vesicles and small vacuoles with secretion are observed in the epithelial cells, mainly close to the plasma membrane (Figure 4E). These vesicles and vacuoles fuse, forming larger structures with electron-dense or electron-opaque secretion (Figure 3E and Figure 4E).

Despite the parietal position of a large quantity of secretory vesicles and vacuoles, there is no sign of their fusion to the plasma membrane to release the secretion. Rare plasmodesmata (Figure 4B) indicate little connection between adjacent epithelial cells, and secretion is only released after lysis of the cell.

An increasing accumulation of electrodense material (Figure 4E), darkening of the cytoplasm (Figure 3A), condensation of chromatin (Figure 2C,D) and rupture of the vacuole and other organelles (Figure 3B) indicate the process of programmed cell death associated with cellulase activity detected mainly in the periclinal cell wall facing the lumen (Figure 2B). This enzymatic activity promotes the loosening of the periclinal cell wall, which becomes shrunken (Figure 3B and Figure 4E) and ruptures, releasing the disintegrated protoplast into the lumen (Figure 1E). The holocrine secretion mediated by programmed cell death and wall digestion of the epithelial cells is also related to the release of reactive oxygen species (ROS) detected by DCFH in some cells during the secretory activity (Figure 4F).

## 3. Discussion

Our study revealed that the resin ducts of *Kielmeyera appariciana* are formed by a schizogenous process and the secretion release is holocrine. The formation of the lumen by cell separation is a complex and poorly explored process that involves digestion of the middle lamella between the initial cells of the duct (rosette) and participation of the cytoskeleton in the polarized growth of the cells that split away. Pectinase activity via loosening of the rosette cells to form an intercellular space has already been detected during the formation of ducts and cavities of other species, such as *Citrus* and *Pinus* [24,25]. In other cases, pectinase and cellulase activities were related to secretion release into the duct lumen, as described in *Protium* [26]. The participation of the cytoskeleton has never been identified during the splitting off of the epithelial cells, but its reorganization to promote diffuse or polarized growth is well-known in several cell types [23,27]. The cytoskeleton arrangement may also be involved in the secretory process, especially in granulocrine secretion, due to its coordination of vesicle trafficking within the cell during exocytosis [28]. A higher quantity of microtubules was observed near the outer periclinal wall of glandular trichomes in *Hyptis* [20,21]. This arrangement, likely related to the release of secretion due to the role of the microtubules and actin microfilaments in the transport of vesicles within the cell, was not observed in *K. appariciana* since there is no exocytosis in the epithelial cells of this species.

### 3.1. Synthesis of Secretion

The resin ducts of *K. appariciana* synthesize mainly terpenes, phenolic compounds, polysaccharides and proteins [19]. The occurrence of these substances is directly correlated with our ultrastructural results, which include the predominance of organelles involved in the production of lipids such as plastids, endoplasmic reticulum and mitochondria [1]. In addition, the presence of dictyosomes and rough endoplasmic reticulum is related to the production of polysaccharides [29]. Similar subcellular characteristics have also been described for resin ducts of various species of Anacardiaceae [30,31,32,33,34,35].

The epithelial cells of the ducts of *Boswellia serrata* also secrete lipids in addition to polysaccharides [36] and the occurrence of glands that secrete exudates of mixed nature is not restricted to ducts of Calophyllaceae. Ducts of various species of Anacardiaceae and Burseraceae, in addition to glandular trichomes of *Inula viscosa* and *Fagonia* and laticifers of all plants, secrete lipids together with polysaccharides and proteins [2,37,38,39,40,41].

The substances produced by ducts, namely resins, polysaccharides or a mixture of both, are highly viscous and have high molecular weights. Therefore, they have some difficulty in freely passing through the plasma membrane and cell wall, which act as a mechanical barrier for the confinement of the synthesized substances inside the cells [1,3]. There are several hypotheses that seek to explain how such substances can be released into the lumen, but the most accepted hypothesis is the explanation that these substances with high molecular weights exert a turgor pressure within the protoplast, pushing the secretion in the periplasmic space to cross the cell wall, reaching the outside of the cell [1,3,42,43,44]. However, the resin of the ducts of *K. appariciana* does not follow this pattern since the secretion does not cross the plasma membrane or the cell wall. Instead, the entire cell disintegrates, releasing the secretion into the lumen (holocrine secretion).

### 3.2. Holocrine Secretion

During the secretory activity, some epithelial cells of *K. appariciana* showed cytoplasm darkening very similar to what happens in the resin ducts of *Mangifera indica* [30]. According to these authors, there is a synchrony in the secretory activity of the epithelium that consists of storing secretion in the periplasmic space and releasing it into the lumen, accompanied by the gradual darkening of the cytoplasm and, finally, cell collapse. It may be the case that these cells do not undergo lysis and remain in the structure of the duct without secretory activity, i.e., in the post-secretory phase, presenting a vacuolated or distorted appearance of the cytoplasm. Cytoplasmic darkening does not happen uniformly in all secretory cells at the same time because the production and release cycles of secretion operate independently in each secretory cell and vary in duration according to the species and the developmental stage of the secretory structure [3,45].

The darkening of the cytoplasm in some cells during the secretory activity of the ducts of *K. appariciana* coincides with the detection of reactive oxygen species, chromatin condensation, vacuole rupture, degradation of organelles and cellulase activity, especially concentrated in the periclinal wall inwards of the lumen. All of these events demonstrate the occurrence of programmed cell death [11,46,47]. Although there have been many studies on ducts and secretory cavities that have programmed cell death as part of their formation [26], this is the first study to show that this highly coordinated process is related to the release of secretion. In the other glands with holocrine secretion, the exudate is released by cell disruption [1,4,5,6] without the gradual and coordinated activity of cell autophagy that culminates in the collapse of the cell.

The secretion mode of oils and resins most commonly described in the literature is eccrine [1,26,43,44,48,49,50,51,52,53]. However, *Kielmeyera appariciana* differs from most of the studies carried out to date since it releases the lipophilic substances produced by epithelial cells through a holocrine process.

Conversely, in the case of mucilage-secreting cells, the secretion is produced by dictyosomes [29,54] and is transferred to the outside of the protoplast via a merocrine process in trichomes of *Ipomea cairica* [55], ducts of *Mangifera indica* [32] and *Lannea coromandelica* [34]. However, the end of the secretory phase in some mucilage-secreting cells is marked by cell death after the cell transfers the mucilage to a large periplasmic space that fills the cell lumen, as observed in the mucilage idioblasts of *Opuntia polyacantha*—Cactaceae—[56], *Araucaria angustifolia* [57] and exotesta of *Euphorbia milii* [58].

## 4. Materials and Methods

### 4.1. Plant Material

Samples of *Kielmeyera appariciana* Saddi were collected on the campus of the Universidade de São Paulo in São Paulo/SP (Brazil) and the voucher was deposited in the herbarium SPF (USP; Costa, E.R. 1). This species was selected based on the previous report of resin ducts in the leaves and young stems that are distinct from those of the secondary stem [19].

### 4.2. Duct Development

Shoot apices were fixed in Karnovsky’s solution [59] for 24 h at 4 °C and postfixed in 1% osmium tetroxide in 0.1 M sodium phosphate buffer pH 7.2. Then, the material was dehydrated in a graded ethanol series, embedded in LR White and serial sectioned at 0.5 µm thickness on a Leica Ultracut UCT (Leica Microsystems Inc., Heidelberg, Germany). Longitudinal and transverse semi-thin sections were stained with toluidine blue and observed under a Leica DMLB light microscope.

### 4.3. Ultrastructure

The samples embedded in LR White were sectioned into 80 nm thick sections and the ultrathin sections were collected on copper grids, counterstained with uranyl acetate [60] and lead citrate [61] and analyzed with a Zeiss EM 900 transmission electron microscope (Carl Zeiss, Oberkochen, Germany).

### 4.4. Pectinase and Cellulase Activities

The detection of pectinase and cellulase activities was based on the protocols of Allen and Nessler [62] and Bal [63], respectively. Shoot apices were fixed in Karnovsky’s solution for 2 h, washed 20 times in 0.1 M sodium phosphate buffer pH 7.2 and stored in the same buffer for 12 h at 4 °C. Part of the material was incubated in 0.5% pectin solution in 0.1 M sodium acetate buffer pH 5.0 and the other part was incubated in 0.02% methylcellulose solution in 0.1 M sodium acetate buffer pH 5.0 for 20 min at room temperature. Later, both materials were transferred to Benedict’s reagent at 80 °C for 10 min and then washed in 0.1 M sodium phosphate buffer pH 7.2. After these treatments, both samples were processed for transmission electron microscopy as usual. The same procedure was applied to the control samples excluding the incubation step in pectin and cellulose.

### 4.5. Programmed Cell Death (PCD)

The investigation of PCD was based on indirect methods of detection of reactive oxygen species (ROS), nuclear chromatin condensation and cytoplasm disintegration. For detection of oxygen free radicals/hydrogen peroxide, free-hand sections of fresh shoot apices were treated with DCFH-DA (2′,7′-dichlorofluorescein diacetate) for 10 min and then washed in deionized water [64]. The sections were analyzed under a Leica DMLB fluorescence microscope equipped with a blue light filter block (excitation filter BP 420–490, dichromatic mirror RKP 510, suppression filter LP 515).

For DNA labeling, samples fixed in Karnovsky’s solution were sectioned free-hand and stained with DAPI (4′,6-diamidino-2-phenylindole; 1 mg.mL^−1^) in phosphate-buffered saline (PBS) and 1% Triton X-100 (Sigma, St. Louis, MO, USA), washed in water and mounted in an antifade mounting medium [65]. The slides were analyzed using a Zeiss LSM880 confocal laser microscope (Carl Zeiss, Oberkochen, Germany) at 405 nm wavelength.

### 4.6. Microtubule Immunolocalization

The protocol of Medina et al. [23] was used for microtubule labeling. Free-hand sections of shoot apices were fixed in PMET buffer (100 mM PIPES, 5 mM EGTA, 1 mM magnesium sulfate, pH 6.9) containing 0.5% glutaraldehyde, 1.5% formaldehyde fixative solution for 40 min. Then, the sections were washed in PMET buffer (100 mM PIPES, 5 mM EGTA, 1 mM magnesium sulfate, 0.05% triton X-100, pH 6.9). An enzymatic cell wall digestion step was subsequently performed using a 0.1% pectinase solution (0.1% pectinase from *Aspergillus aculeatus* Sigma-Aldrich, 0.4 M mannitol, 1% BSA and 1× PBS) for 20 min at room temperature. After rinsing off the pectinase solution with PMET buffer, the sections were incubated for 3 h in the permeabilization buffer (PBS, 1% Triton X-100 in 1× PBS, pH 7.5) at room temperature. After washing in PBS, sections were incubated in a sodium borohydride solution (1 mg/mL sodium borohydride in 1× PBS) and then transferred to blocking buffer (1% BSA, 50 mM glycine in 1× PBS).

All sections were incubated in a 1.5 mL Eppendorf tube with a 1:1000 solution of the monoclonal mouse anti-α-tubulin antibody, clone B 512 (Sigma-Aldrich catalog number T6199) in blocking buffer at 4 °C overnight. Five washes of 10 min each in the incubation buffer (50 mM glycine in 1x PBS) were carried out and then the sections were transferred to the blocking buffer for 30 min. After that, the samples were incubated in a 1.5 mL Eppendorf tube with the secondary antibody in a 1:100 solution of Alexa 488-conjugated goat anti-mouse IgG antibody (Invitrogen catalog number A28175) in a blocking buffer for 3 h at 37 °C. Samples were then washed in PBS and mounted in an antifade mounting medium [65]. The sections were analyzed using a Zeiss LSM880 confocal laser microscope (Carl Zeiss, Oberkochen, Germany) at 405 nm wavelength. DAPI was also used as a counterstain for microtubule analysis.

## 5. Conclusions

Our study opens new horizons for understanding the involvement of hydrolytic enzymes and the cytoskeleton in the process of schizogenous formation of secretory ducts and holocrine secretion release. This secretory process, although uncommon since it promotes the death of cells, derives from programmed cell death, releasing the resin of *Kielmeyera* into the lumen. PCD related to the release of secretion with the involvement of reactive oxygen species and cell wall digestion is described for the first time in a plant gland and may be a key innovation of Calophyllaceae.

## Data Availability

All figures and tables in this manuscript are unpublished and were generated specifically for this article.
